# Individual-Based Modelling Potentials and Limitations

**DOI:** 10.1100/tsw.2002.179

**Published:** 2002-04-19

**Authors:** Broder Breckling

**Affiliations:** University of Bremen, Center for Environmental Research and Technology (UFT), Section 10: Ecology, Leobener Strasse, D-28359 Bremen, Germany

**Keywords:** individual-based models, self-organisation, potentials, limitations, technical aspects, applications, arthropods, fishes, plants, ecosystems research, agentbased models, development perspectives

## Abstract

Individual-based modelling (IBM) is an important option in ecology for the study of specific properties of complex ecological interaction networks. The main application of this model type is the analysis of population characteristics at high resolution. IBM also contributes to the advancement of ecological theory. One of the remarkable potentials of the approach is the possibility of studying self-organization and emergent properties that arise from individual actions on higher integration levels, especially on the population level.

This review outlines the background and different application fields of individual-based models together with a short description of the technical implications of model setup. The limitations of this modelling approach result from the technical basis of model construction, which can handle a limited number of active entities only. Limits in biological knowledge also restrict the application of this model type. The paper presents some individual-based models that have been developed for different purposes and briefly discusses these models. Concerning the perspective of IBM, a coincidence with developments in artificial life research is explained. IBM shifts the focus of ecological analysis of dynamic systems from structurally fixed settings to the analysis of self-organizing interaction patterns that are variable in quantity and quality.

### BACKGROUND AND EMERGENCE OF INDIVIDUAL-BASED MODELLING

Individual-based modelling (IBM) is a topic that has been receiving rapidly increasing attention in ecology for more than 10 years. The introduction of IBM extended the set of available modelling techniques. Before the 1990s, the differential equation–based approach was widely dominant in ecological model applications. Differential equation models were employed to study population dynamics and processes of energy and biomass turnover in food webs in particular.

This approach is well suited to describe homogeneous populations in homogeneous environments where the state of the individuals is similar enough and their number high enough to characterise the population as a whole in terms of continuous variables. However, differential equations are of limited use if the heterogeneity of either the environment or the response and the characteristics of the individual entities are too high to be reasonably described in terms of qualitatively invariant variables that only differ in quantity over time[1,2]. To extend the spectrum of available techniques and to deal with situations where the standard equation-based approach is limited, a range of different modelling techniques has emerged:

- Fuzzy set models that can deal with vague and imprecise information;
- Neural nets for complex optimisation or pattern recognition tasks;
- Knowledge-based systems to organise information about specific ecological topics;
- Geographical Information Systems, which allow various options to overlay and analyse spatial information.

For each modelling technique there is a certain range of applicability for ecological purposes that allows researchers to deal with tasks that cannot be solved in a comparable way with other approaches.

We find that individual-based models are applicable and recommendable if a model must deal with details of biological information about organisms like their specific behaviour, activity, development, and interactions. Individual-based models allow one to study how the state and actions of the involved organisms contribute to specific properties on the integration level of the population. This approach allows the emergence of particular characteristics of the higher integration level as a result of "atomistic" relations and processes on the lower level to be analysed. Because they deal with factors such as the state, position, and action of single organisms (or ecological entities), individual-based models operate on the basic level of ecological consideration. They do not require aggregation of biological information in the form of averages. In this regard the IBM approach provides a different strategy for achieving generality compared to differential equation models: differences among the individuals are not leveled out. They are taken into consideration as bases of the interactions. Individual-based models therefore allow analysis of the dynamics of the distribution pattern of features, characteristics, and attributes of the considered organisms in a homogeneous as well as heterogeneous context [1,2].

A main definition criterion for an individual-based model is that the organisms under consideration are represented not only as countable entities, but also as organisms with one or more additional features that specify the particular state of the individual. The degree of resolution that the description of an individual is to be extended — whether it consists of a number of state variables only, or also contains instructions to process the variables, or contains information concerning connections to other individuals, or whether the model includes representations of environmental structures — is a matter of choice and depends on the problem to be dealt with[3]. In this respect, individual-based models vary greatly. Most of them are implemented in general-purpose programming languages. However, in recent years, some specialised modelling tools for IBM became available.

The first examples and conceptions of individual-based models emerged during the 1970s. One of the first ecologists working in this field was Kaiser[4,5]. He used SIMULA, the first object-oriented programming language. His models represent individuals with a number of different states, like location and physiological status, together with instructions to modify the variables according to the context that the modeled organism finds itself in. Other early works are from Hogeweg[6,7] and Hogeweg and Hesper[8], who also used SIMULA in the beginning. DeAngelis et al.[9] must also be mentioned among the pioneers in this field.

Individual-based models were later established as a new category of ecological models. Den Boer[10] in 1979 and Lomnicki[11] in 1988 point to the conceptual reasons for why, in many cases, the development of a population can be understood only if the underlying interactions are brought down to the individual level. Huston et al.[12] showed in 1988 that IBM provides an approach that extends the scope of ecological modelling to problems that conventional models cannot cope with. The object-oriented programming technique[13,14,15], which extended the options in model construction of IBM in a considerable manner, became more widely known among ecological modelers[16]. In 1992, DeAngelis and Gross[17] presented a widely accepted description of the state-of-the-art in IBM. In 1994, Judson[18] summarised the first phase of the wider application of IBM. Also in 1994, DeAngelis et al.[19] discussed strategic issues regarding the approach. A later review was given by Grimm[3] in 1999.

Based on the application experience that has been achieved to this point, in the following section we specify the application fields where models that consider the individual level are preferred. We then turn to technical aspects such as how to construct an individual-based model. The next section describes some examples of applications. Finally, we discuss the limitations and the development potential of IBM.

### POTENTIALS

A special feature of IBM is that the description level of the model is very close to the information level that results from empirical investigation. Field ecologists frequently can map their observations 1:1 to model properties. This feature has advantages as well as drawbacks, and it is important to outline the potential of this approach. On one hand, the setup of an IBM requires less mathematical effort in many cases. The model tells its story by means of computer code. On the other hand, it is usually not possible to separate the model from the computer code and present it in the form of a few equations. The advantage of qualitative precision implies a higher effort to achieve intersubjectivity because it increases the effort needed to understand a model that someone else has written. The topics listed below give an impression of which types of ecological interactions are frequently dealt with in IBM applications; in most cases, IBM include more than one of the aspects discussed here.

#### Representation of Heterogeneous Environments

When one is striving to understand the role of spatial heterogeneity in ecological systems it is useful to represent spatial relations in a spatially explicit model. In many cases this is of crucial importance to understanding the success or failure of organisms in particular environments. Representations of the environment can take different forms and involve different degrees of complexity. Some models use only simple geometrical forms (e.g., squares and circles), which are sufficient to study colonisation frequencies or migration patterns [see 2,20,21]. Others include complex structures that are highly realistic and result from Geographical Information Systems or remote sensing data[22,23]. Spatial explicitness requires the model organisms to evaluate spatial information for orientation or to adapt their behavioural repertoire.

#### Orientations of Individuals, Behavioural Patterns, and Actions of Individuals

To make the behavioural repertoire context-specific, the modeled individuals need to detect the type of surrounding they find themselves in. Specification of movement and orientation is a topic that is frequently analysed in individual-based models. While orientation always includes the retrieval of external information, the modeled organisms usually specify their actions due to a combination of external impact from their environment and their internal state (e.g., whether they are hungry, find themselves in a reproductive phase and so on). The specification of what an organism will do in which kind of situation defines its behavioural pattern. How specific this description needs to be depends on the intention of the model. It can range from simple stepwise movement rules[20] to a model description of motion, energetics, and complex interaction patterns[23]. Any of these descriptions require a case-specific diagnosis and evaluation of the organisms' situation. The considered actions can therefore be selected, parameterised, or modified according to the specific context[1]. In elaborated cases this specification can take the form of a small expert system that connects current input from the modeled environment to the adequate response of the individual in the model.

### Interaction of Individuals

If the interaction of single organisms is represented and investigated in a model, the use of an individual-based approach is implied. Specific properties of the modelling environment are required for this purpose. Usually interaction partners are not the same throughout the simulated time. Therefore it is useful to allow the variable establishment of references (pointers) between organisms as well as references to other objects (e.g., environmental structures like nesting sites) and to specify which one of a potentially large number of objects is the particular partner to which an interaction process is applied[1,2,22]. One frequently used operation in IBM is the detection of nearest neighbors[24,25], or, in trophic interaction, the detection of prey individuals or prey densities close to the position of the predator that allows an adaptation of the movement direction[26]. The range of interactions that can be modeled is limited only by the ability of the programmer and his or her tools.

### Population Self-Structuring Processes

Provided that the model specification is complete in terms of its description of relevant environmental aspects, the behavioural pattern, and the terms of interaction, an individual-based model can be used to investigate a wide range of self-structuring processes on the population level. The description of actions on the level of single organisms in the model leads to particular consequences concerning population development. This is why IBM is an excellent way to study emergent properties[2,19,22]. Concerning ecological theory as well as applications this is one of the most exciting aspects that can be exemplified — especially when one considers the following topics.

#### *Cannibalism*

Cannibalism occurs in some predatory populations, and cannibalistic activities can change the size structure and age structure of the population[15]. Such activities can be involved in an adaptation process to environmental fluctuations, for example in periods of food shortage. Cannibalism is found in a wide range of different taxa. As it implies an individual-to-individual relation, an IBM approach is required to discover how probabilities of cannibalistic events and the size relations of the involved organisms influence the population structure. The model output can be compared with an empirical distribution. The model can help to decide whether the assumptions about the process are in accordance with the overall observations. Examples of such an analysis are given by DeAngelis et al. in 1979 for largemouth bass[9], and by Dong and DeAngelis in 1988 for smallmouth bass[28].

#### *Schooling*

Schools are self-organised aggregations of a few up to hundreds of thousand individuals. As a whole, schools exhibit behavioural strategies that differ from those that isolated individuals perform, for example concerning orientation in an environmental gradient[2,25]. How this self-organisation process takes place is an exciting topic for IBM. It is possible to observe schools in the field (i.e., the outcome of the interaction on the higher integration level), while the description of the underlying mechanisms allows for alternative behavioural details to be considered. Fish schools were modeled by Huth and Wissel[24], Reuter and Breckling[25], and Romey[29]. In contrast to empirical studies, it is possible to test the model assumptions under arbitrary conditions. It turns out that approaches that provide a reasonable description in homogeneous situations may fail to describe schooling if a heterogeneous environment is implemented in the model. Fish schools are frequently studied in two-dimensional models. A three-dimensional approach inspired by birds, but remaining on an abstract level of a movement and self-organisation study, has been termed *boids.* A comprehensive collection of information on this approach is available on the Internet at http://www.red3d.com/cwr/boids/.

#### *Ant Trails*

Ants are frequently used as a topic of IBM. They are well suited for illustrating self-organisation and aggregation phenomena that are based on individual-to-individual interactions. In most cases the modelers are interested in the organisation of the movement pattern and the foraging success when searching spatially dispersed food items[21,30]. How can an ant colony optimise foraging success under the condition that no individual has the complete overview and perceives only a very limited part of the environment? The individuals perform a random search and mark their path with pheromones. When they tend to follow paths with higher pheromone concentrations there results an aggregation on the colony level that represents a “higher order.” Due to this self-organisation effect all ants together acquire a largely different foraging success than the sum of the same number of independently searching individuals. Probably because of the simplicity of the individual action, which usually focuses only on movement and not on the complete ant biology, the topic is used as a paradigm for agent-based modelling outside biology in computer science and robotics.

#### *Self-Thinning of Plants*

Interactions that lead to a decrease of differences between individuals can usually be dealt with on the level of averages. However, interactions that increase initial differences may lead to the development of pronounced distribution spectra in the population. Mutual shading of plants is such a process. An individual in an even-sized plant stand that gained a small advantage over a competitor by chance may extend the discrepancy because improved light access allows accelerated growth, which can cause a more pronounced advantage[31,32]. To understand intra- and interspecies interactions, it may be necessary to represent dynamic plant architectures as well[33,34,35].

### Interaction of Different Components of the Ecological Context

We have already mentioned the realism of individual-based models, which implies that the range of interactions that can be represented in a model is not necessarily limited to one main topic. Using IBM, it is possible to study how different characteristics of an organism, which may change during its ontogenetic development, influence the overall result of the performance of the population in a realistic or hypothetical context. This encourages scenario investigations that can stimulate assumptions on how a population responds in new environments, or how a context would change if particular individual characteristics were altered. This leads to a kind of sensitivity analysis that does not only register the quantitative change of an output variable as a result of changed inputs, but also extends this idea to qualitative or structural changes. To execute this kind of study, which is valuable especially in survival studies of endangered species, one must include an almost complete description of the relevant organismic properties — especially behaviour and energetics, and environmental characteristics. Models of this type are described for example by Fleming et al.[36] and Wolff[23] for wading birds, Reuter and Breckling[22] for the European robin, Comiskey et al.[37] for the Florida panther and the white-tailed deer.

### IBM Approach in Other Disciplines

The concept of IBM is not exclusive to ecology. The representation of individuals and their actions is also used in other disciplines where it is relevant to study the development of networks of active entities that influence each other mutually or that interact with a structured environment. Under the name of *agent-based simulation* or *complex adaptive systems* (CAS) technical systems are studied with the same modelling approach. Interactions of computer networks as well as decision-making robots and artificial life simulations use widely related techniques. In fact, in this field the inspiration of biological and technical applications is mutual. The Web site http://www.santafe.edu/projects/echo/echo.html presents John Holland's simulation environment, "Echo," which focuses on the self-organisation of physical entities. An extension of this approach by Ginger Booth, known as "Gecko," is presented on the World Wide Web at http://peaplant.biology.yale.edu:8001/papers/swarmgecko/rewrite.html. While artificial life considerations help to describe properties of real life in models, it can also be asked how the operation of technical entities can be designed to mimic characteristics of organisms in a general-systems context. In this regard, individual-based models are one example of CAS.

### TECHNICAL ASPECTS OF MODEL SETUP

There is no unique way to specify IBM; various options exist. Among those we describe two approaches in coarse terms: list-oriented and object-oriented.

The list-oriented approach can easily be implemented in almost any programming language, but is restricted to comparatively simple application cases. The considered states of the individuals (e.g., age, biomass, or other physiological properties) are arranged in the columns of a table in which each individual takes one row. The row describes its state (i.e., the individual is represented by a state vector). The model requires an updating routine that calculates successive states of each individual. For example, growth can be calculated according to a context-specific growth function that takes into account spatial positions or specific condition factors. In a simple way, even interactions can be modeled: if the individuals have coordinates to indicate their positions, it is possible to search nearest neighbors. They could be considered as prey (or mates) depending on what the model deals with. The limitation of this approach is the fact that the updating code is the same for all list elements. The more case-specific the modelling task is, the more difficult the model is to maintain.

For complex interactions the second approach is more adequate. Object-oriented programming and simulation is currently used for most IBM applications. It requires the employment of an object-oriented programming language like Simula, Smalltalk (ObjectWorks), C++, Delphi, or Java. These languages provide code elements that combine storage space for variables with instructions to change (or update) the variables. These elements — usually called *classes* — can be copied or deleted during program execution so that during runtime an increasing or decreasing number of similar structured units can be concurrently active, each with its own unique specification. The interaction of a large numbers of objects requires a coordination scheme. In most cases, the user is responsible for its organisation. The programming language Simula was the first to provide an elegant scheme to handle this task in the background: Each object sends a message to a central instance, at which point in simulation time it requests an update. This is called an *event*. The update consists of an execution of the internal code of the particular object. It may (and usually will) generate a new update request, or event, at a later point in time. The instance in the background receives the requests from all objects, stores them in an event list, and keeps the list sorted according to the time axis. The execution control is then passed to the object that generated the first event in the list and deletes the event after the update. Execution then proceeds to the next object in the list. In this way it is possible to handle large numbers of concurrent processes in a quasiparallel manner. Parallel processing is mimicked by sorting events, which are scheduled at different points on the time axis. If events are scheduled for the same point in time by different objects, the order of entry in the event list determines the updating sequence. In this case updating is done without incrementing the time value. This object scheduling procedure is called *discrete event*

*simulation*[7,8]. A detailed introduction is given by Pooley at the Web page http://www.cee.hw.ac.uk/~rjp/bookhtml/; see chapter 19 in particular.

Operating on this background, each object consists of a set of variables that describe the state of an individual, a set of pointers that are used to establish (variable) connections to other objects, and a number of statements that specify the actions of the individual after the instantiation. The central part is a compound loop statement that is repeated as long as the individual is operating. We call it the *life-loop*: As long as a Boolean variable "alive" is true, the condition for a repeat is given. One run of the loop updates the variables due to the specific conditions of the object encounters (considering internal as well as relevant external conditions). Additionally, the time increment is calculated when the next update cycle takes place and the respective event is generated[1]. A scheme of the functioning is given in Fig. 1. This short sketch of program structures can be refined to meet a wide range of biologically relevant situations that are dealt with in individual-based models. The application examples presented in the following section use this construction scheme.

### APPLICATIONS OF IBM

Examples 1 to 3 below introduce applications for a variety of different organisms (beetles, fishes, plants, etc.) and topics (movement, dispersal, growth, light competition, etc.). They exemplify how the scheme given in Fig. 1 can be used to deal with widely different ecological problems. In addition, Example 4 outlines a complex model network for a number of different species. For each example I list references to further information sources.

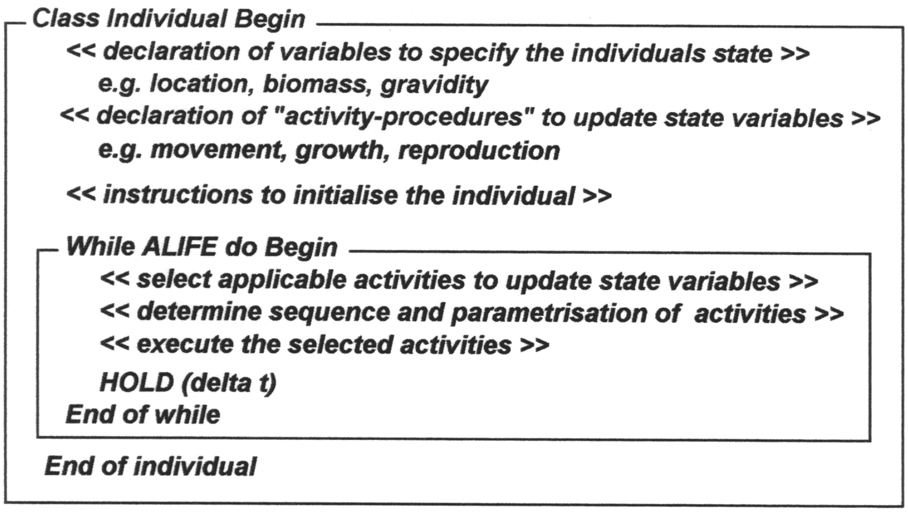

**FIGURE 1.** General structure representing an individual using an object-oriented modelling approach: The class specifies the general structure. During simulation, various copies (objects), which act as independent entities, can be started. The internal loop is a central data structure that is repeated as long as the object is considered "alive." This loop updates the state variables of the objects according to the applicable instructions for the specific situation and the specification of the interval until the next updating round ("Hold"). One of the activities can be reproduction (i.e., starting an object of the same type).

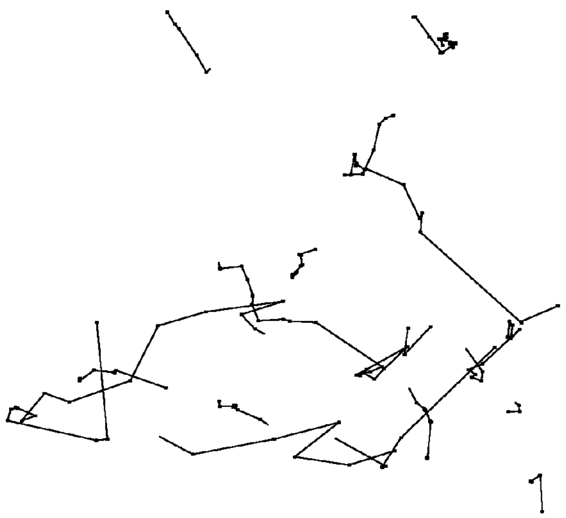

**FIGURE 2.** Telemetrically recorded movement pattern of the ground beetle *Carabus clathratus*. Each polygon represents subsequent daily positions of an individual. (Fred Jopp, unpublished data).

### Example 1: Movement—Tracks of Individuals

In the field, movement activities of many organisms can be observed only for short time periods. If these observations are sufficient to specify a set of rules, then a model can be specified. The model can make projections concerning dispersal processes for longer time intervals and larger areas than those observed. An example is the modelling of ground beetle dispersal. For several beetle species there are records of successive daily positions for up to a few weeks: Baars, who used radioactively labeled beetles[38]; Hockmann et al.[39,40], Charrier et al.[41], and Riecken and Ries[42], who used radio tracking. These data allow compilations of the spectrum of distances and angles that mark the end points of a day's movement activity. Frequently, there is no pronounced correlation in the angles of subsequent daily positions, so in the model they can be chosen at random. The distances moved can be approximated by an inverse distribution: The larger the distance between two subsequent daily positions, the more rarely it occurs—and the shorter a distance, the more frequently it is found. The empirical distribution statistics can be used directly for a model specification. Running the model allows one to investigate how changes in the movement pattern affect long-term distribution[20,43]. Refinements of this approach may include temperature dependencies or responses to spatial heterogeneities. From the model results we can learn that rare events in movement activity can be very meaningful for long-term distribution patterns. It turns out that distribution distances tend to be underestimated if only average distances are used (see Fig. 2, Fig. 3A–C).

**Additional sources on the Internet** — Byers[44] has dealt with the type of functions that underlie this approach. The paper can be accessed at http://www.wcrl.ars.usda.gov/cec/papers/ecol01.htm.

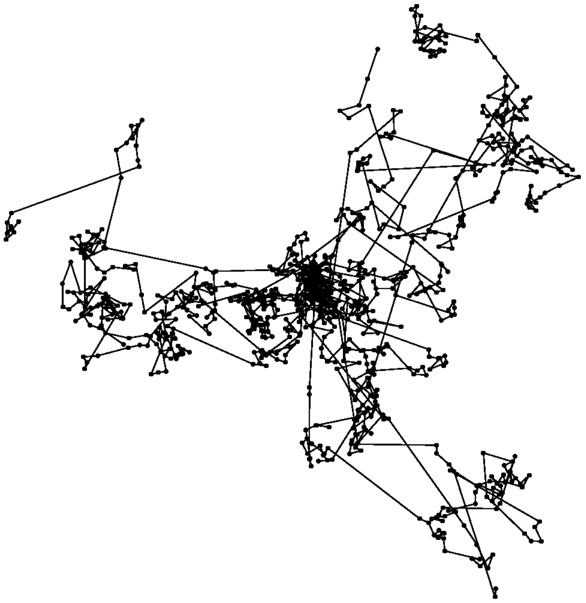

**FIGURE 3A.**

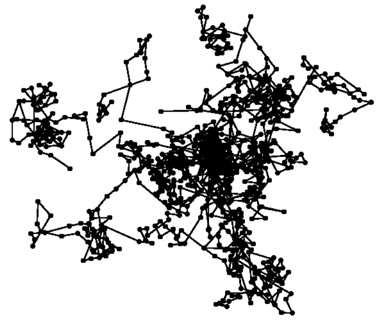

**FIGURE 3B.**

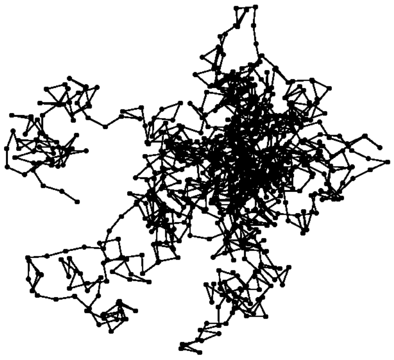

**FIGURE 3C.**

**FIGURE 3.** Based on data of Riecken and Ries[42] for the ground beetle *Carabus coriaceus,* a dispersal model was adapted[20,43]. To demonstrate the consequences of the movement specification for the distribution pattern, ten model individuals were started at the center of the area (representing 300 by 300 m) over a period of 90 days conforming to one annual activity period. (A) The standard model using the step range distribution of the observation; (B) 5% of the largest daily steps were eliminated from the available randomly chosen step sizes; (C) an average step size was used for each day instead of a spectrum of different step sizes.

### Example 2: Physiological Differentiation — Dynamics of Size Spectra in a Fish Population

The foraging success of individuals is one of the key determinants of their biomass development. Food searching in a heterogeneous environment can involve a random component. If the size of an organism itself feeds back positively to foraging success, an increase of initially small differences may occur. This was demonstrated for cohorts of roaches: *Rutilus rutilus*, a species occurring in many temperate freshwater habitats[26,45]. The study site was Lake Belau, in Northern Germany. Roaches prefer to stay in the central part of the lake during daytime and during the night. Only at dawn do they move to the shallow littoral areas where food abundance is higher. This can be interpreted as a predator avoidance strategy. Since swimming speed is correlated to body size, larger individuals need less time on average to reach favourable sites during the random movement they exhibit. Starting with an evenly sized cohort, a characteristic length distribution emerges that can also be observed in the field. Frequently, growth variations are attributed to genetic differences. Model studies of this type allow the contributions of different factors to be distinguished. In this case we can see the extent to which habitat structure in context with behavioural repertoire contributes to biomass differences in the absence of genetic differences (see Fig. 4, 5). Apparently, a lake's size and its form are important in explaining the size distribution pattern. For this purpose the model must represent and connect the factors of movement, growth, and energetics together with the heterogeneous habitat structure.

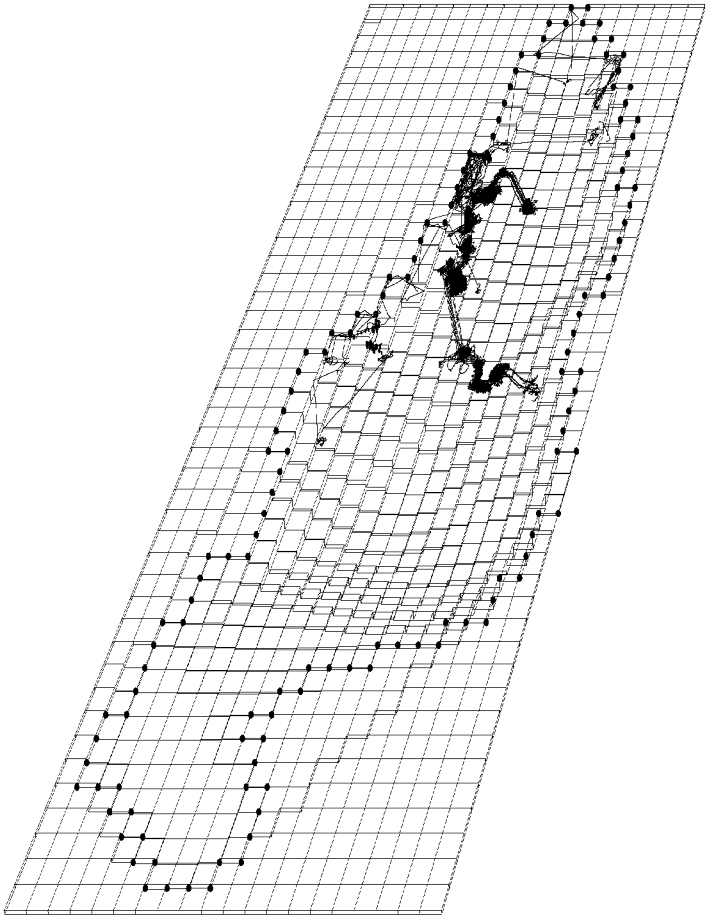

**FIGURE 4.** Tracks of ten roaches (*Rutilus rutilus*) in Lake Belau (Northern Germany) during a time period of 3 days. The simulation environment was derived from a GIS map. Each cell represents a 50- by 50-m surface. At night the swimming activity of the roach is reduced. The fish form loose schools if even-sized individuals are within sight range. In the model, the position is updated in 5-min intervals.

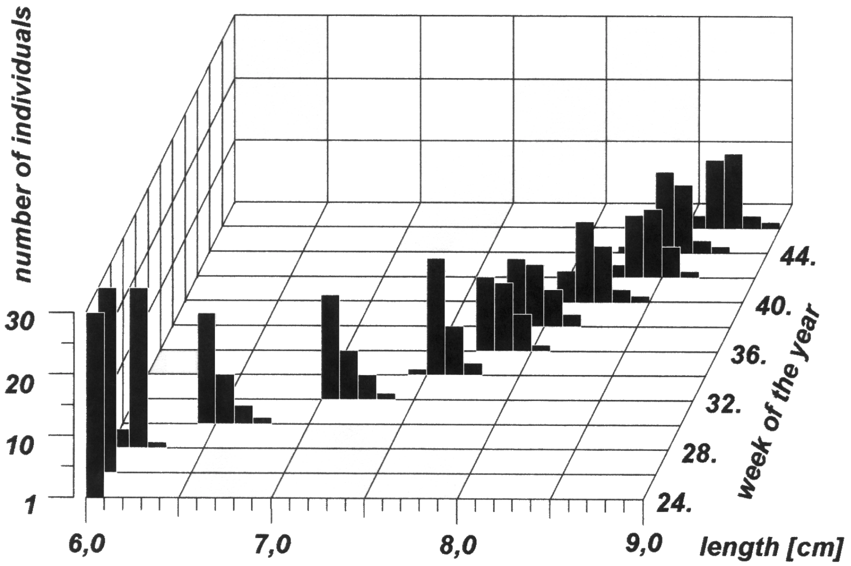

**FIGURE 5.** Simulation output for the length differentiation in a cohort of 30 young roaches. The simulation starts with individuals of 6 cm in length. Due to the movement pattern and the environmental structure of Lake Belau (see Fig. 4) a positive feedback results, which increases minimal random differences and leads to a characteristic size spectrum even if there is no genetic variability involved.

**Additional sources on the Internet** — A good compilation of references of fish population studies, including a number of IBM approaches, is available at the following address: http://www.esd.ornl.gov/programs/COMPMECH/comp_pubs.html.

### Example 3: Structural Development of Modular Organisms — Plant Modelling

The modelling of modular organisms has to meet specific requirements. For many ecological questions it is not sufficient to represent the organism as a structurally invariant entity, but one must model the dynamics of module formation and its adaptation to the particular environmental context. This applies not only to higher plants but also to some groups of animals like bryozoa, corals, and sponges. For the latter, Kaandorp[46] provides a variety of modelling results. Until now, plant modelling has been predominantly discussed in the context of botany, agronomy, and forestry rather than in an interdisciplinary context of IBM. A main development trait is the description of plant architecture[47,48,49,50,51]. Representing dynamic interactions of modules requires special effort. The L-systems approach of Prusinkiewicz and Lindenmayer[52] uses rule-based, recursive rewriting of strings and their graphical interpretation to model the successive development of the architecture of a plant. Kurth[53] extended this approach to simulate context-sensitive features.

Of the large number of available plant models, we present the Almis model here[33,34,35]. It works with an object-oriented approach and allows a context-sensitive simulation of structural development as well as an integration of the underlying physiological processes. In its present form it is parameterised for alder trees (*Alnus glutinosa*). The model includes internal processes for each module type (e.g., internodes, leaves, roots, meristems; see Fig. 6). Each module operates and adapts its own photosynthetic activity, storage, and transport of assimilates and nutrients according to its particular internal conditions and the conditions of its environment. If rules are specified as to how a module responds to any of the potential situations, the development of the whole organism and interaction network on the plant-stand level emerges as a result of self-organisation. The model

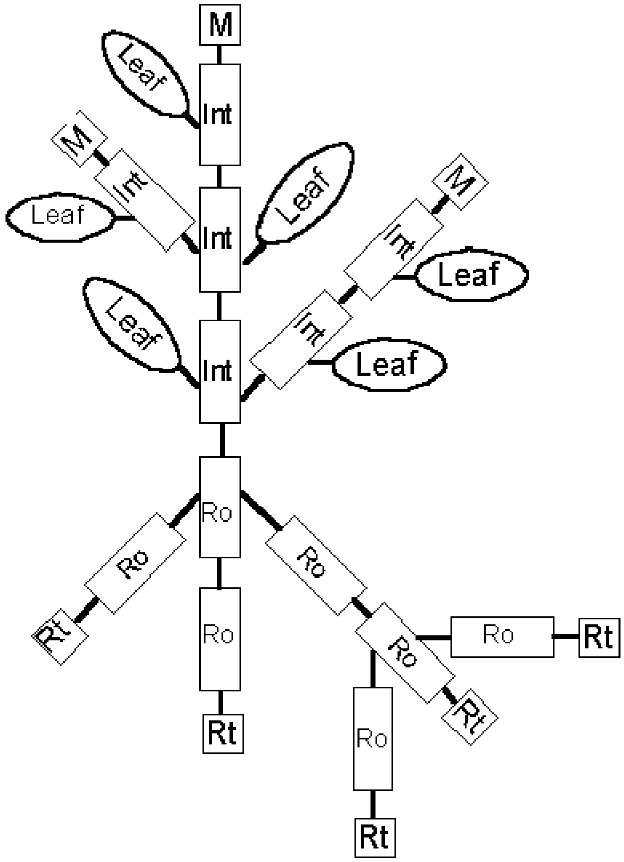

**FIGURE 6.** Modules in the black alder (*Alnus glutinosa*) Almis model. Each module (internode, leaf, meristem, root, roottop) runs its own processes, such as assimilation, transport, growth, and start of new modules. The shape of the whole plant emerges context-specific as a result of the module activities.

allows conclusions about the long-term implications of physiological measurements that provide information on short-term responses of particular plant units (Fig. 7). It may be interesting to compare this approach with that of Ford and Diggle[32] and Ford and Sorrensen[31], which focus on light competition and describe the process on a more integrative and less detailed basis.

**Additional sources on the Internet** — There is abundant information on plant modelling available through the Internet. A group headed by Prusinciewicz[47,52] at the University of Calgary, Canada, developed a complex modelling environment under the name of "the Virtual Laboratory" and publishes its results at http://www.cpsc.ucalgary.ca/Research/ (under "biological modelling and visualisation" and under "graphics jungle") and http://www.cpsc.ucalgary.ca/Redirect/bmv/index.html. The French institution CIRAD, where de Reffye and others[49,50,51] developed the Amap approach, provides access to scientific aspects of their work at http://www.cirad.fr/presentation/en/program-eng/amap.html, and makes software products available at http://www.bionatics.com/. The plant modelling group headed by Kurth[53] is working at the University of Göttingen, Germany (http://www.uni-forst.gwdg.de/~wkurth/). A majority of plant modelers belong to the Plant Architecture Information System (http://pais.cirad.fr/index.html), which offers a list of contacts as well as a bibliography (http://pais.cirad.fr/bibliography.html).

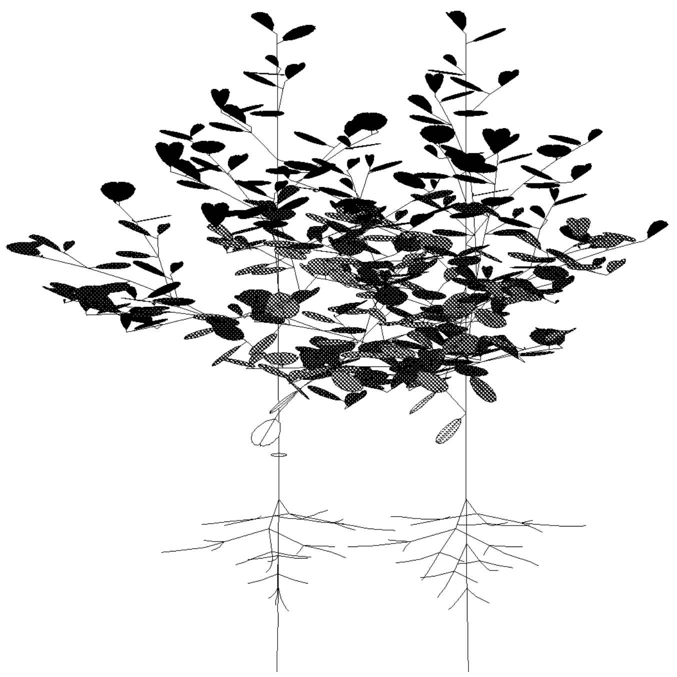

**FIGURE 7A.**

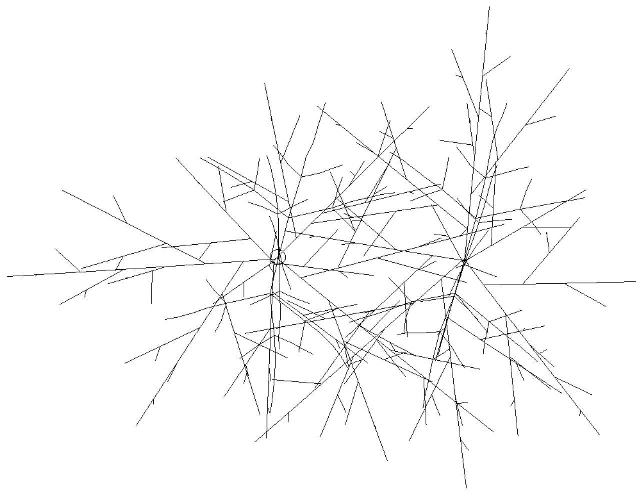

**FIGURE 7B.**

**FIGURE 7.** Graphical representation of a model run that simulates competition for light of two young alder trees. Different light exposure of leaves due to mutual shading leads to different amounts of assimilant gain (indicated by grey shading of leaves). As a result of these differences local structural adaptations of the plant architecture occur due to different local growth rates. A horizontal view and a vertical view of the shoot (without leaves) are shown.

### Example 4: IBM in a Complex Application Network

Models for particular species usually cover only a comparatively narrow focus of the overall ecological context. When individual-based models operate on the landscape level, they usually represent the environment as the home range for the focal organisms rather than executing an ecosystem study. For management purposes it can be desirable to include the larger ecosystem network. To combine both aspects — the analysis of the most interesting species and the wider ecological context — it is useful to develop IBM as a part of a model network. The ATLSS (Across Trophic Level System Simulation) project is a well-recognised example of this approach. The project's goal is to provide integrated management recommendations for the Everglades National Park in Florida, U.S.[54,55]. For this purpose a combination of different modelling techniques was employed, working together in order to cover the spectrum of different scales, spatial ranges and resolution requirements. The ATLSS project uses Geographic Information System (GIS), grid-based models operating with differential equations, and — especially for focal organisms on higher tropic level — individual-based models. Focal organisms modeled on the individual level are endangered species such as wading birds, the seaside sparrow, the snail kite, and the Florida panther as well as the white-tailed deer. All of the models integrate a large amount of empirical data. Without modelling, it would be difficult to understand the overall outcome of the interdependencies of the numerous components. Fig. 8 lists the models involved. The approach is fruitful in several respects. It shows the application relevance of modelling, enables an interdisciplinary cooperation with a strong ecological focus, and provides valuable experience in the methodology necessary to operate complex models[56].

**Additional sources on the Internet** — The ATLSS project is well documented at the projects Web site (http://www.atlss.org/), which offers publications, technical reports, and examples of model output. When searching for other application examples, modelling groups and foci of interest concerning IBM it may be useful to consult Craig Reynolds annotated list of links of individual-based models at http://www.red3d.com/cwr/ibm.html. This list covers various application fields including biological and technical (e.g., traffic, multimedia) references.

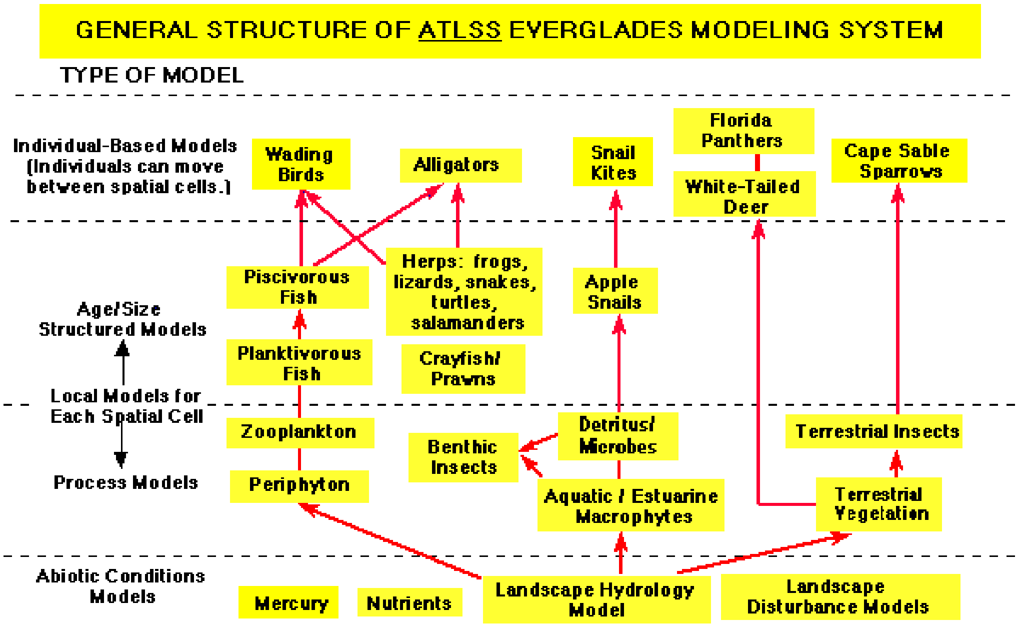

**FIGURE 8.** Overview of the different modelling topics and model types used in the ATLSS project (from http://www.atlss.org/ ).

## LIMITATIONS

IBM extends the range of topics that can be dealt with in a formal way. To specify the application range, we must discuss the limitations that are encountered. Below we list the main limiting factors.

### Parameter Limitations

When setting up an individual-based model, the first limitation that developers usually face is the problem of *parameterisation.* An individual-based model does not operate on the level of averages. Thus it is not possible to overcome lack of information by using coarse generalisations and abstraction. It is necessary to come up with precise and — even more difficult to achieve — complete descriptions of what an organism does in any relevant situation it meets. Therefore, a high level of requirements concerning biological information has to be met for practical applications. If theoretical principles are investigated, the parameter specification is not that challenging. Hence individual-based models are developed mainly for organisms that are well studied in the field. In this context, IBM is very helpful to answer the question of whether the basis of empirical information is complete or whether more investigations are needed to adequately describe the performance of the considered entities.

Modelers whose main experience is in modelling on the population level may tend to overestimate this kind of limitation. The parameters required for the description of an individual are not abstract, but rather are conceptually accessible. There is usually a certain empirical range of parameter variation. Another aspect should be noted in this context. In individual-based models most of the parameters tend to be rather insensitive, while on the population level the functions may be governed by parameters where extremely small changes affect results dramatically. Individual entities need to be comparatively robust. Without this relative insensitiveness, individuals would not be able to cope with a changing and variable environment. The organism needs a certain buffer potential. This means that high precision is not required for most of the parameters.

### Capacity Limitations

The number of individuals that can be represented in a model is not infinite. Even though it is possible to use the facilities of object-oriented programming languages to automatically generate and delete units that represent individuals, storage capacity limits have to be taken into consideration. Depending on the number of variables, the length of the code used for each object, and the hardware constraints for practical applications, it is currently the case that individual-based models are restricted to dealing with no more than a few hundred thousand individuals in simple cases. Complex applications can usually deal with much smaller numbers only. This is enough for many applications, but may restrict others.

Capacity limitations do not occur with respect to storage only: Increasing the number of individuals usually increases execution time. Certain types of interactions increase execution time exponentially with the number of individuals. This is the case for some types of searching procedures in which each organism determines its nearest neighbor and compares its own coordinates to the coordinates of all others. To some extent it is possible to expand the limits by measures like running the model on faster computers. It is therefore useful to work with a programming environment that is not hardware-specific and that allows the model to run on other machines and under other operating systems. Additionally, in many cases a considerately economic program design can help to solve capacity problems. One technique in this regard is the proposition of Sheffer et al.[57] to aggregate organisms that are similar according to the question under consideration to one “super individual.”

Any technical solution extends capacity limitations only relatively. There are relevant ecological questions that can be investigated with IBM only in a restricted way. Fish schools of several tens of thousands individuals are one example, trees with hundreds of thousands of leaves and branches are another. It is not always the case that extremely large numbers of individuals interact in a trivial way that allows an aggregated model representation.

#### Connectivity and Hierarchy Limitations

The description on the individual level can be considered a basic level in ecology. Higher integration levels (e.g., population, ecosystem, and landscape levels) can be obtained as aggregations of the involved lower levels. Individual-based models therefore are a means to study the connection of different hierarchical levels. It must be noted that this is possible only to a limited extent. It is currently not possible to operate a complete ecosystem model on the level of individuals, except perhaps for rather simple cases in theoretical considerations (e.g., Booth, http://peaplant.biology.yale.edu:8001/papers/swarmgecko/rewrite.html). It is difficult to bridge large-scale spectra. For example, it may be possible to handle the dynamics of bacterial decomposers individually on a micro scale. But within the same model resolution, it seems rather unlikely to be able to simulate processes on the landscape level. The landscape level may be adequate for dealing with larger organisms. Connecting different hierarchical or scale levels to a larger extent is possible to a limited extent. To deal with scale phenomena, it is necessary to work with additional upscaling techniques that have been developed in geostatistics. On a lower level, processes may be simulated on an individual base, and the result when aggregated and extrapolated may be passed to the next higher level (e.g., habitat-use probabilities). Individual descriptions are therefore mainly tools to describe single species — or selected interactions of members of a few populations.

### DEVELOPMENT PERSPECTIVES

The special contribution of IBM is its ability to formalise the modelling of organisms with a wide range of different types of interactions and to observe the resulting emergent phenomena. We expect that IBM will continue to bring to light implications of particular aspects of individual behaviour. Generic approaches usually cover certain abstract aspects of organisms. What is interesting, however, is the potential of IBM to cover the full range of case-specific structural and functional variations. In this regard, predefined simulation tools need to be judged according to the extent to which the freedom of the modeler to change the underlying conception to meet the requirements of any specific case is restricted. The freedom of a general-purpose programming language should not be given up in favour of a narrowly limited toolbox concept. In many cases the development of a model from scratch will probably not be more time-consuming than the adaptation of a library package. This may be the reason why many modelers working in an empirical biological context tend to use general programming languages (e.g., [22,23,25]). In the technical context for the simulation of abstract complex systems interactions, specialised simulation packages may be preferable: Take for example Swarm, which was developed at the Santa Fe Institute. This simulation environment was developed in the artificial life context; see Hiebler (1994) at http://cam.cornell.edu/~hiebeler/swarm-paper.html, or, for current developments go to http://www.swarm.org/. Another example is Ecotools, which was developed in connection with the University of Oldenburg, Germany; see Vogel et al. (2000)[58] or http://www.offis.uni-oldenburg.de/projekte/ecotools/. Plant-modelling tools were mentioned in Example 3 above (see the plant architecture information system at http://pais.cirad.fr/index.html).

Concerning ecological applications, the research focus for studying the transition from one integration level to the next is one of the most interesting perspectives. The study of emergent properties is a core topic. IBM is an integrative instrument for advancing ecological theory[1,12,17] as well as for developing practical ecological handling guidelines and serving planning purposes in species protection and in landscape management[36,54]. IBM has an increasingly important aspect as a tool for population studies useful for analysing protective measures for endangered species in variable environments and for analysing self-organisation and evolutionary strategies. Ecology will continue to benefit from individual-based models as they are able to describe the connection of behaviour, energetics, and heterogeneous structures.

### Artificial Life

In a methodological sense, there is a development tendency in IBM towards more complex action patterns for the entities described. Ranging from a relative minimum of describing an organism with a few variables to describing complex models of behaviour and interaction, there is an obvious link to applications outside biology. The principles that are used to specify the model of an organism can be equally well employed to describe an abstract, autonomously acting entity. This means that there is a methodological overlap among ecological modelling, robotics, simulation of technical systems, and simulation of sociological interactions. A recent development is including intentional behaviour, learning, planning, and anticipation in autonomously acting entities. This is one interesting focus of artificial life studies. Artificial life research emerged as a well-recognised topic at the Santa Fe institute (http://www.santafe.edu/). The Alife conference (http://alife.org/), originally initiated by the Santa Fe Institute, is a main source of information where this interdisciplinary connection is promoted.

### Shifting the Focus of Systems Analysis

Concerning the implications of model structures, we are witnessing a transition of paradigms in ecology in which IBM is largely involved. The classical dynamic models, which focused on systems with a fixed structure, attempted to describe the dynamics in terms of quantitative changes of certain variables. The advanced individual-based models open up a new perspective. They can simulate not only changes of quantity, but can also show how the structure of a system changes as a result of internal causes. This is due to the possibility of starting new objects that represent additional individuals (or terminating current ones), and of allowing relations between objects to change as a result of the interaction. This implies a new perspective in systems analysis towards a wider spectrum of dynamics that involves quantitative as well as qualitative transitions. The perspective possibility to investigate the combination of structural, functional and quantitative dynamics is one of the most challenging options in Eco. Model.
